# Re-Patterning Sleep Architecture in *Drosophila* through Gustatory Perception and Nutritional Quality

**DOI:** 10.1371/journal.pgen.1002668

**Published:** 2012-05-03

**Authors:** Nancy J. Linford, Tammy P. Chan, Scott D. Pletcher

**Affiliations:** 1 Department of Molecular and Integrative Physiology, Geriatrics Center and Nathan Shock Center of Excellence in the Basic Biology of Aging, University of Michigan, Ann Arbor, Michigan, United States of America; 2 Department of Human and Molecular Genetics, Huffington Center on Aging and Developmental Biology Program, Baylor College of Medicine, Houston, Texas, United States of America; University of Pennsylvania, United States of America

## Abstract

Organisms perceive changes in their dietary environment and enact a suite of behavioral and metabolic adaptations that can impact motivational behavior, disease resistance, and longevity. However, the precise nature and mechanism of these dietary responses is not known. We have uncovered a novel link between dietary factors and sleep behavior in *Drosophila melanogaster*. Dietary sugar rapidly altered sleep behavior by modulating the number of sleep episodes during both the light and dark phase of the circadian period, independent of an intact circadian rhythm and without affecting total sleep, latency to sleep, or waking activity. The effect of sugar on sleep episode number was consistent with a change in arousal threshold for waking. Dietary protein had no significant effect on sleep or wakefulness. Gustatory perception of sugar was necessary and sufficient to increase the number of sleep episodes, and this effect was blocked by activation of bitter-sensing neurons. Further addition of sugar to the diet blocked the effects of sweet gustatory perception through a gustatory-independent mechanism. However, gustatory perception was not required for diet-induced fat accumulation, indicating that sleep and energy storage are mechanistically separable. We propose a two-component model where gustatory and metabolic cues interact to regulate sleep architecture in response to the quantity of sugar available from dietary sources. Reduced arousal threshold in response to low dietary availability may have evolved to provide increased responsiveness to cues associated with alternative nutrient-dense feeding sites. These results provide evidence that gustatory perception can alter arousal thresholds for sleep behavior in response to dietary cues and provide a mechanism by which organisms tune their behavior and physiology to environmental cues.

## Author Summary

Sleep is a fundamental biological process regulated by evolutionarily conserved molecular mechanisms. In this work, we demonstrate a novel link between gustatory perception of sugar and sleep patterning in *D. melanogaster*. The presence of low dietary sugar reduced the arousal threshold for waking, leading to repartitioning of sleep into a larger number of episodes throughout the day. Gustatory perception was both required and sufficient for this effect. Further addition of sugar to the dietary environment suppressed the effects of gustatory perception through a gustatory-independent mechanism. Although the quantity of dietary sugar also regulated fat accumulation, gustatory perception was not required, indicating that diet-induced changes in obesity and sleep behavior may be mechanistically separable. These findings illustrate a mechanism for the regulation of behavioral state by the availability of dietary nutrients through the interplay between gustatory and non-gustatory factors.

## Introduction

Sleep is a fundamental biological process regulated by conserved molecular mechanisms [1]. Understanding how sleep behavior is regulated by environmental inputs can provide key insights into disorders of sleep and the basic mechanisms underlying the responsiveness of organisms to environmental stimuli.

When an animal is completely deprived of nutrients, a starvation-associated foraging state is initiated that is characterized by sleep loss and elevated activity [2], [3]. However, dietary nutrient quantity within the nutritionally sufficient range can also regulate the health status of an organism, including effects on obesity [4], immunity [5], and lifespan [6]. While total caloric content plays a role, there are important indications that specific nutrients exert effects independent of the total calorie intake [7]. Therefore a deeper understanding of how changes in the quantity of available dietary nutrients can regulate behavior and physiology is required.

While there are many clues suggesting that dietary factors can influence sleep, the relationship between diet and sleep has not been clearly defined. In human populations, obesity is associated with sleep dysregulation [8]. Furthermore, the sequence of satiety in mammals and invertebrates is marked by the cessation of eating behavior and a subsequent immobility or sleep behavior [9], [10]. Finally, in mammalian systems, shared pathways exist through orexinergic neurons for the regulation of metabolism and sleep behavior [11]. It is likely that an evolutionarily ancient mechanism exists to regulate an interaction between sleep and the dietary environment.

We have characterized the relationship between sleep behavior and dietary nutrient availability in *Drosophila melanogaster*. We find that dietary sugar, but not protein, provokes a change in the partitioning of sleep episodes without affecting total sleep or waking activity. This change is sleep behavior is associated with modulation of the arousal threshold for waking. We have uncovered an interaction between gustatory sensory perception and metabolic factors, which underlies the effects of dietary sugar on sleep behavior. These results provide key insight into how an organism interprets its dietary environment in order to modulate behavior.

## Results

### Dietary Sugar Modulates Sleep Architecture

**Figure 1:**
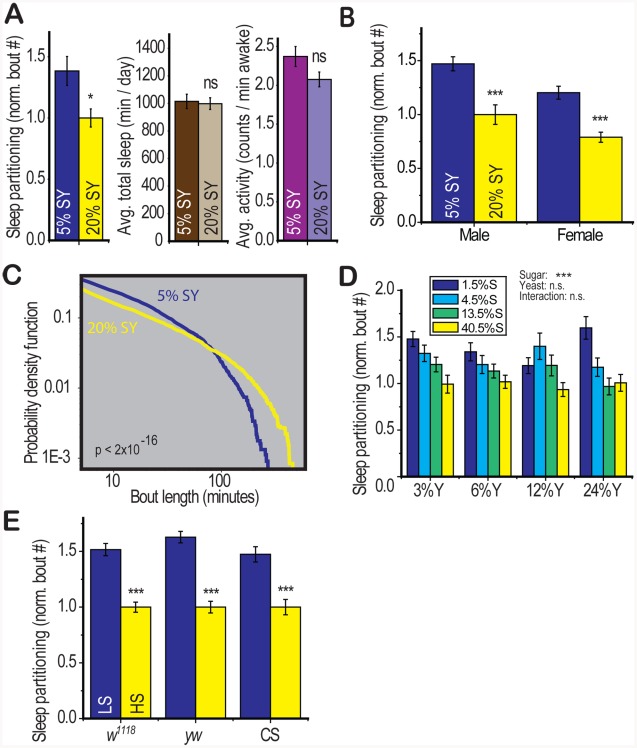
Dietary composition modulates sleep architecture. (A) Control (*yw*) flies fed 5∶5% sucrose∶yeast (5% SY) displayed an increase in the number of sleep bouts (sleep partitioning) over a 5 day recording period relative to those on 20% SY food. Total sleep and average waking activity (beam crosses per minute) were unaffected. (B) The response to diet was present in both males and females. (C) The distribution (probability density function) of sleep bout lengths over the same 5 day recording period revealed that the 5% SY diet resulted in a reduced number of long sleep bouts in favor of a larger number of short and medium-length bouts (p<2×10–16, Kolmogorov-Smirnov test). (D) The carbohydrate (sucrose) component of the diet modulated sleep (p<0.001) with no significant effect of yeast or sucrose∶yeast interaction (two way ANOVA). Concentrations of sucrose (S) and yeast (Y) are shown as percentages by weight. (E) The effects of diet were robust in three control genotypes: *w^1118^*, *yw*, Canton S (CS) when flies were fed on a base of 2.5% yeast with either low (LS, 2.5%) or high sucrose (HS, 30%). Error bars represent mean +/− SEM for each group. *** p<0.001, ** p<0.01,* p<0.05 for all statistical calculations. Significance values for t-tests between dietary conditions are shown above each set of bars and significance values for two-way ANOVA are shown above the graph, where applicable. See Figure S1 and Tables S1 and S2 for additional information.

We conducted a behavioral screen for diet-induced phenotypic changes using conditions known influence *Drosophila* lifespan (5% sucrose∶yeast compared to 20% sucrose∶yeast) [6]. Diet robustly altered sleep architecture, increasing the number of sleep bouts during both day and night without altering total sleep, activity, or latency to sleep (Figure 1a, Table S1) in both males and females (Figure 1b). Analysis of the distribution of sleep bout lengths revealed that a low nutrient diet led to a reduction in the number of long sleep bouts while increasing the number of short and medium-length bouts (Figure 1c, p<2×10^−16^ Kolmogorov-Smirnov test). We also analyzed the distribution of sleep across the day and found few diet-induced changes except for a slight shift toward an earlier onset of daytime inactivity under low nutrient conditions (Figure S1a) that did not translate into a consistently significant change in the daytime latency to sleep (Tables S1 and S2). Thus dietary factors specifically regulate sleep by modulating the partitioning of sleep episodes throughout the 24-hour circadian period. *Drosophila* sleep is a well-characterized behavior that involves prolonged periods of inactivity (sleep bouts) in a stereotyped position with reduced sensitivity to environmental stimuli [12], [13]. It is a useful model for identification of factors that may have relevance to human sleep behavior.

*Drosophila* food is composed of a protein source (Brewer's Yeast) and a carbohydrate source (sucrose). By separately modulating dietary yeast and sucrose across a range of physiologically relevant concentrations, we determined that dietary carbohydrate (p = 2×10^−8^) was the source of diet-induced sleep partitioning and neither yeast nor the carbohydrate∶yeast interaction were significant in a two-way ANOVA model (Figure 1d). We modulated only dietary carbohydrate for further experimentation.

To further address the effects of dietary sugar on sleep behavior, we identified a dietary paradigm (LS = 2.5% sucrose and HS = 30% sucrose in a 2.5% yeast base medium) that mediates changes in health status (longevity) within the single-fly activity tube environment used in these studies (Figure S1b). We observed a robust effect of dietary sugar on sleep partitioning in three control genotypes with no other consistent behavioral changes (Figure 1e, Table S2), similar to the effects of modulating sugar and yeast together (Table S1). We further observed that baseline sleep patterns are altered by environmental factors including food preparation method and early post-eclosion housing density [14], but there is no interaction between the effect of diet and the effect of environment (Figure S1c). Furthermore, examination of the relationship between diet-induced sleep partitioning and the total number of sleep episodes for multiple control experiments conducted across a variety of environmental conditions revealed no significant correlation (Figure S1d). We also verified that the effect of dietary sugar on sleep partitioning was robust in the presence of a water source (Figure S1e) and on sucrose-agar medium without yeast, which is a standard medium for behavioral analysis (Figure S1f) [15].

Together, these results indicate that diet-induced sleep partitioning is robust across multiple strains and environmental conditions and is a generalizable phenomenon that may have relevance to mammalian sleep response.

**Figure 2:**
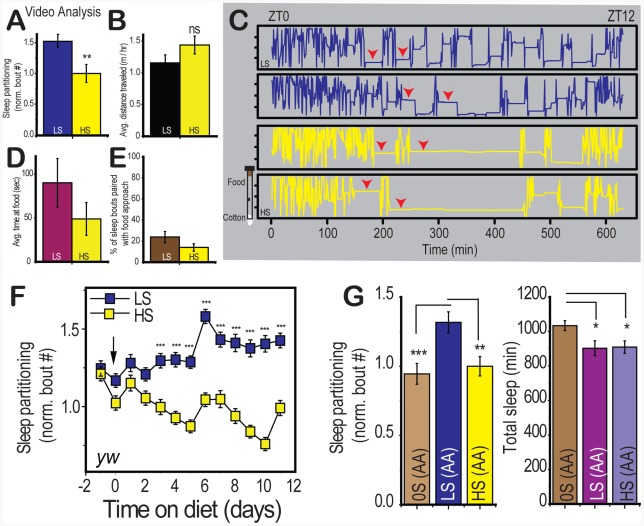
Characterization of diet-induced changes to sleep architecture. (A) Video tracking analysis confirms the effect of sugar on sleep bout number was significant when the position of individual control (*yw*) flies was tracked with video monitoring (n = 15 per group). (B) The average distance traveled per hour was not significantly different between dietary conditions. (C) Position traces along the length of the food vial are plotted across time for two individuals per dietary condition.Arrows indicate the presence of sleep episodes. The food is oriented at the top and the cotton plug is at the bottom in this visualization (see graphic to the left of the plots) (D) The average time spent in a trip to the food and (E) the percent of sleep bouts resulting in an approach to the food within two minutes were not significantly different between dietary conditions. (F) Control flies were switched from 10% sucrose∶yeast fly medium to LS (2.5% sucrose) or HS (30% sucrose) in a 2.5% yeast base medium on day zero and sleep was measured for the subsequent 12 days. Flies were switched to fresh food on day 6. Each point represents the avergage +/− SEM for a 24 hour period.(G) Control flies were placed on a yeast-free amino acid mixture containing either 0%-OS(AA), 2.5%-LS(AA), or 30%-HS(AA) sucrose in order to compare complete sugar deprivation to the low and high sugar condition on a consistent sugar-free base medium. Error bars represent mean +/− SEM for each group. *** p<0.001, ** p<0.01,* p<0.05 for all statistical calculations. Significance values for t-tests between dietary conditions are shown above each set of bars. For panel C, significance values were calculated by one-way ANOVA followed by Fisher LSD for comparison between groups. See Figure S2 for additional supporting evidence.

We and others have used an infrared beam-based activity monitoring system (Trikinetics DAM system) to study *Drosophila* sleep [16], however detailed tracking analysis can provide additional information about the precise location of the fly relative to the food source and rule out the possibility that feeding behavior may be mimicking sleep. We confirmed the effects of diet on sleep partitioning using video tracking in the activity tube environment (Figure 2a, 2c – arrowheads indicate sleep episodes) and also determined that the distance traveled was unaffected by dietary conditions, as expected from analysis of activity levels in the DAM system (Figure 2b). As previously reported [17], *Drosophila* sleep occurs away from the food source regardless of dietary sugar content (Figure 2c), making it infeasible that feeding itself could mimic sleep behavior in response to dietary shift. The average duration of trips to the food source was also unaffected by dietary conditions (Figure 2d) and substantially shorter than the five minute threshold for considering an inactive period a sleep episode. We have also measured the number of rare trips to the food that exceeded five minutes and found no significant difference between groups (5.2% (LS) or 5.5% (HS) of feeding bouts exceeded five minutes in length, p = 0.938, t-test), making it impossible for trips to the food to mimic the reported changes in sleep behavior. To test whether motivation to approach the food (“hunger”) may provoke early awakening from sleep under low nutrient conditions, we examined the number of sleep bouts followed by an approach to the food source within two minutes and found no significant difference between dietary conditions (Figure 2e), indicating that the drive to feed was not directly causing an interruption in sleep. Instead, we conclude that the organization of sleep behavior throughout the day represents a behavioral state that reflects an organism's ability to perceive concentration differences in specific dietary nutrients.

Changes in sleep partitioning were first observed within 24 hours of dietary manipulation in two control genotypes, and the differences continued to increase until stabilizing at 96 hours (Figure 2f, Figure S2a). These results indicate that the effects of diet are rapid and sustained and that they likely reflect a diet-induced change in the animal's internal physiological state rather than a transient response to the dietary shift. Furthermore, the effects of diet are completely reversible when the diet is switched from LS to HS or HS to LS (Figure S2b). This result supports the assertion that the dietary conditions are not causing damage but instead evoking a sleep state through modulation of a signaling pathway.

We next wished to determine whether the presence of dietary sugar was required for the sleep behavior observed on low nutrient food. Low sleep partitioning in the absence of sugar would indicate that dietary sugar is sensed by the organism as an initiating stimulus for re-patterning sleep behavior. We used a yeast-free amino acid base medium (Piper and Partridge, personal communication and [18]) that supports survival without added sugar (AA) and determined that the number of sleep bouts on 0% sugar (0S) food is comparable to the effects of high sugar and that low sugar induces sleep partitioning above baseline levels (Figure 2g, left panel). This result demonstrates that the presence of dietary sugar is required for the sleep partitioning observed on LS food. While we did observe a slight increase in total sleep on 0S food (Figure 2g, right panel), we observed no change in total activity or progressive sleep loss under the 0S conditions used (Figure S2c, S2d), indicating that the 0S medium is not inducing either a starvation-associated foraging phenotype or general sickness. Our observations are consistent with the conclusion that sleep partitioning is a specific phenotypic response to low dietary sugar. These initial results support a preliminary model involving dietary sugar as both an initiator of sleep partitioning and as a suppressor at high concentrations.

### Diet-Induced Sleep Partitioning Does Not Depend on Circadian Rhythm or Known Sleep Regulators

**Figure 3:**
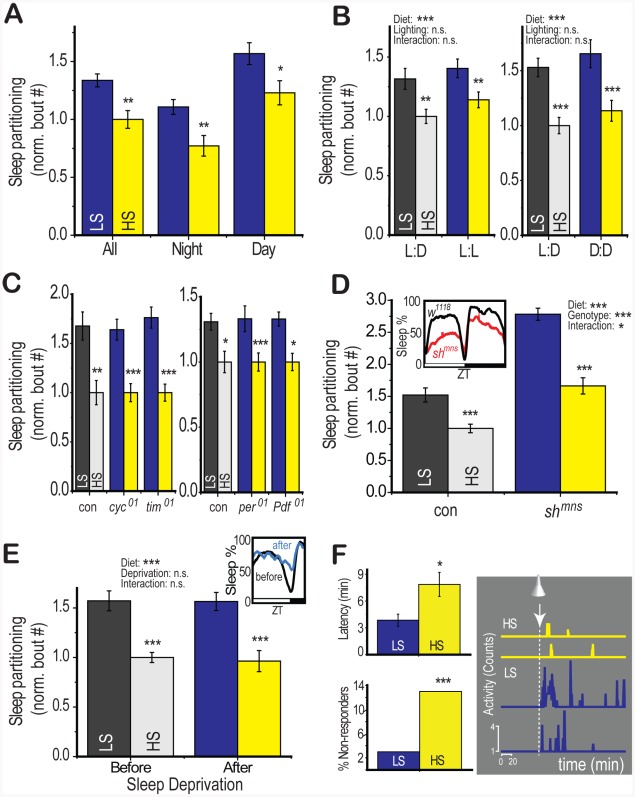
Diet-induced changes to sleep architecture persist despite disruption of circadian rhythm and total sleep. (A) Modulation of dietary sucrose content to 2.5% (LS) or 30% (HS) in a low (2.5%) yeast base increased sleep partitioning during both the day (lights-on) and night (lights-off) period during a 12∶12 hour light∶dark circadian cycle. (B) Disruption of circadian light cues with constant darkness or constant light for 5 days following a period of 12∶12 hour light∶dark entrainment did not disrupt the effects of diet on sleep (no significant effect of diet∶lighting interaction by two-way ANOVA). (C) Dietary sugar modulated sleep partitioning even in the absence of the circadian clock components *cycle* (*cyc^01^*), *timeless* (*tim^01^*), *period* (*per^01^*), and the circadian neuropeptide *pigment dispersing factor* (*Pdf^01^*). (D) Mutants containing the *shaker minisleep* (*Sh^mns^*) variant showed low total sleep relative to genotype-matched controls (inset). However, the *Sh^mns^* flies retained the ability to respond to dietary sugar. A significant diet∶genotype interaction (two-way ANOVA) reflects an exacerbation of diet effects by the *Shaker* mutation (E) Sleep deprivation was induced by one night (12 hours) of a shaking stimulus after 4 days on LS or HS food in control (*yw*) flies. During the 12 hour period following the deprivation, total sleep was increased relative to the identical period prior to deprivation (inset). A significant effect of diet persisted across conditions and there was no significant diet∶deprivation interaction (two-way ANOVA). (F) Flies were stimulated with a 5 minute light pulse 1 hour after lights-off during a 12∶12 hour circadian cycle. This is a time when >80% of flies are typically within a sleep bout and only flies exhibiting sleep at the time of the light pulse were used for analysis. LS-fed flies showed reduced latency to activity from the onset of the light pulse (upper left panel) and increased activity following the light pulse (2 representative traces from single flies, right panel). The number of flies showing no activity within 3 hours following the light pulse but resuming activity the following day (non-responders) was significantly higher in the HS-fed condition (Fisher's exact test). Error bars represent mean +/− SEM for each group. *** p<0.001, ** p<0.01,* p<0.05 for all statistical calculations. Significance values for t-tests between dietary conditions are shown above each set of bars and significance values for two-way ANOVA are shown above the graph, where applicable. See Figure S3 for additional supporting evidence.

We wished to determine more precisely how diet affected sleep by examining its epistatic interaction with known sleep regulators. Sleep is modulated by circadian systems that regulate the timing of sleep as well as homeostatic systems that regulate total sleep amount and respond to sleep deprivation [19]. In *Drosophila*, sleep behaviors occur during day and night, although sleep is primarily consolidated during the dark phase (night) of a 12∶12 hour light∶dark cycle. We observed LS-induced sleep partitioning during both day and night in a 12∶12 hour light∶dark cycle (Figure 3a, Tables S1 and S2). This response persisted in constant darkness and constant light, with no significant effects of the lighting regime or interaction between diet and lighting in a two-way ANOVA model (Figure 3b).

The persistence of a diet response in constant light, an environmental stimulus that rapidly suppresses circadian rhythm (Figure 3b), indicated that effects of diet would be independent of circadian rhythm. Indeed, mutants lacking the core circadian clock components *cycle* (*cyc^01^*), *timeless* (*tim^01^*), and *period* (*per^01^*), or the circadian neuropeptide *pigment-dispersing factor* (*Pdf^01^*) exhibited a normal diet response (Figure 3c), demonstrating that diet interacts with sleep regulatory centers through a pathway that does not require the circadian clock.

Sleep regulators such as the minisleep *shaker* channel variant (*Sh^mns^*) disrupt the regulatory mechanism controlling total sleep, leading to a short-sleeping phenotype, an increased number of sleep bouts, and an altered response to sleep deprivation [20]. We found that LS diet further increased the number of sleep bouts in *Sh^mns^* (Figure 3d), demonstrating that the effects of diet on sleep partitioning are not mediated through pathways involving the *shaker* channels. Instead, we note that the diet-induced sleep response appears to be exacerbated in the short-sleeping mutant (two-way ANOVA p = 4×10^−10^ for diet, p = 2×10^−7^ for genotype, p = 0.002 for interaction). This may represent a synergistic effect between sleep loss-mediated and diet-mediated pathways for modulating sleep architecture. We have also confirmed this result using an alternative method for generating sleep loss. Ectopic expression of an activating ion channel (TRPA1) in octopamine neurons induces sleep loss and elevates the number of sleep bouts [21]. We observed a similar synergistic enhancement of diet effects by induction of sleep loss using this system (Figure S3). We also performed the opposite experiment by experimentally elevating total sleep levels. In this case we used one night of sleep deprivation to evoke an increase in sleep on the following day (sleep rebound). Despite an observable increase in total sleep during the rebound period (Figure 3e, inset), the diet-induced sleep partitioning response remained intact (Figure 3e, two-way ANOVA p = 2×10^−6^ for diet, p = 0.8 for deprivation, p = 0.3 for interaction), suggesting that activation of sleep rebound is also not sufficient to suppress the effects of dietary shift. We propose the existence of a novel sleep center that responds to dietary cues to regulate sleep partitioning without evoking sleep loss. This regulatory center is independent of circadian rhythm and the mechanisms regulating total sleep amount.

### Dietary Sugar Modulates Arousal Threshold

Given that dietary sugar modulated sleep partitioning throughout the 24-hour circadian period rather than during a specific window of the day, we suspected that diet may be directly targeting endogenous mechanisms for regulating the arousal from the sleep state. A reduction in the set point for sleep depth would increase the probability of waking at any point and thereby lead to an increase in the number of sleep bouts across the 24-hour circadian period. Such a change in sleep depth would also alter the response to a wake-inducing stimulus. We tested arousal from sleep in response to a wake-inducing light pulse and found that a larger proportion of LS-fed flies responded to the light (Figure 3f) with more activity (Figure 3f) and a shorter latency than HS-fed counterparts (Figure 3f). This result indicates that dietary sugar may be directly targeting brain centers involved in the regulation of sleep depth or the mechanism of transition between the sleeping and waking state. We note that the latency to sleep following the nightly lights-off transition was not significantly affected by diet (Tables S1 and S2), indicating that diet probably does not interact with sleep initiation. These results support the conclusion that diet modulates sleep partitioning by specifically targeting the arousal threshold for waking without modulating waking activity.

### Diet-Induced Sleep Partitioning Is Independent of Fat Accumulation

**Figure 4:**
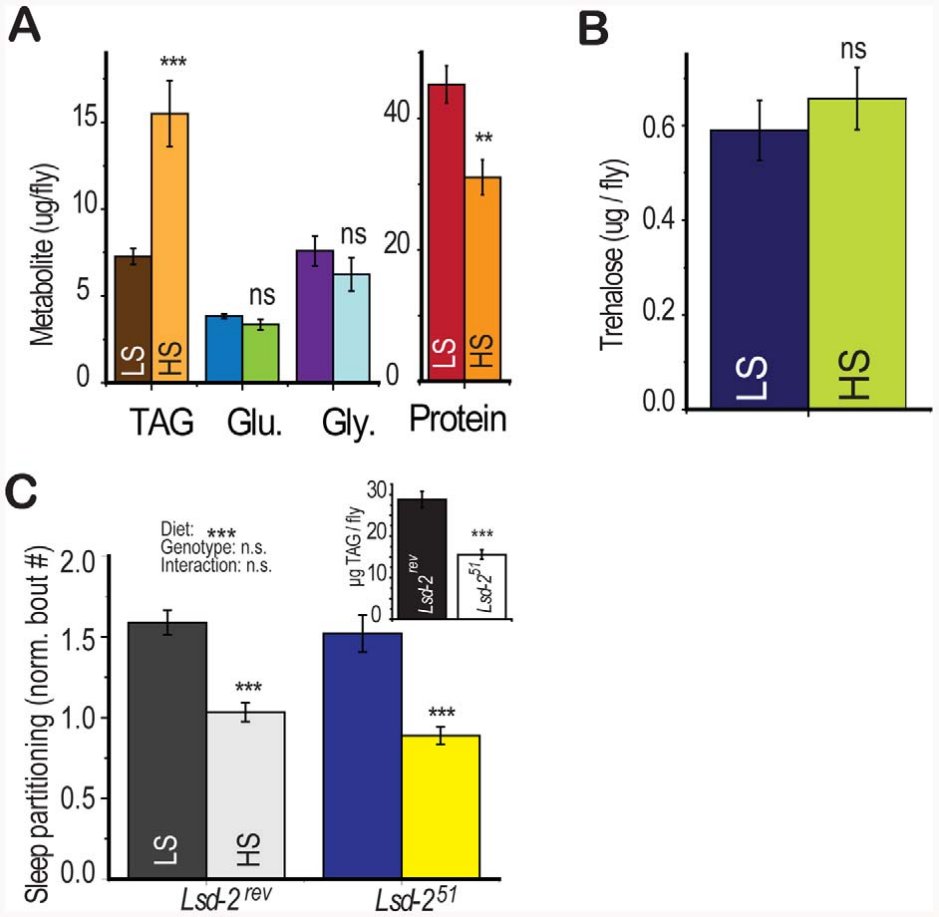
Diet-induced shift in energy storage is not sufficient to induce sleep partitioning. (A,B) A metabolite panel was tested in control (*yw*) flies that had previously been fed 2.5% (LS) or 30% (HS) sucrose on a 2.5% yeast base food for 6 days during activity monitoring. There was a significant change in triglyceride (TAG) and a smaller change in protein levels with no significant effect on glycogen (Gly), glucose (Glu), or trehalose. (C) Flies carrying the imprecise P-element excision deletion *Lsd-2^51^* had reduced triglyceride∶protein levels relative to precise excision *Lsd-2^rev^* controls (inset) but retained a significant sleep response to diet with no diet∶genotype interaction (two-way ANOVA). Error bars represent mean +/− SEM for each group. *** = p<0.001, ** = p<0.01,* = p<0.05 for all statistical calculations. Error bars represent mean +/− SEM for each group. *** p<0.001, ** p<0.01,* p<0.05 for all statistical calculations. Significance values for t-tests between dietary conditions are shown above each set of bars and significance values for two-way ANOVA are shown above the graph, where applicable.

In addition to the effects of dietary sugar on sleep architecture, we also observed a rapid diet -induced shift in energy storage characterized by increased triglyceride accumulation and slight protein depletion [4] without a change in glucose, glycogen, or trehalose levels (Figure 4a, 4b). Dietary protein is known to strongly regulate protein translation and whole-organism protein content [4], [22], [23] but did not modulate sleep behavior (Figure 1d). However, we did not know if total triglyceride levels may directly induce sleep partitioning. To test this possibility, we manipulated fat storage genetically, using flies mutant for the perilipin homolog *Lsd-2* [24]. *Lsd-2* mutant flies are lean and contain roughly 50% of stored triglycerides compared to control animals (Figure 4c, inset). However, there was no significant effect of genotype on the number of sleep bouts, despite a substantial effect of diet (Figure 4c, two-way ANOVA p = 4×10^−10^ for diet, p = 0.2 for genotype, p = 0.6 for interaction). These results establish that low triglyceride levels alone are not sufficient to induce sleep partitioning.

### Gustatory Perception Is Required for Diet-Induced Sleep Partitioning

Gustatory inputs serve as a primary sensor for assessing the sweetness of a sugar source, allowing the organism to make feeding decisions based on predicted nutritional content. *Drosophila* gustatory receptors are localized primarily on the labella but also in the pharynx, tarsi, wing margins, and ovipositor [25]. Within each taste sensillium, the response to sugars is mediated by neurons expressing the *Gr5a* trehalose receptor and the paralogous *Gr64a-f* cluster, which is required for an appetitive response to multiple sugars but not fructose [26]. We hypothesized that these inputs may be important for the behavioral response to dietary cues.

**Figure 5:**
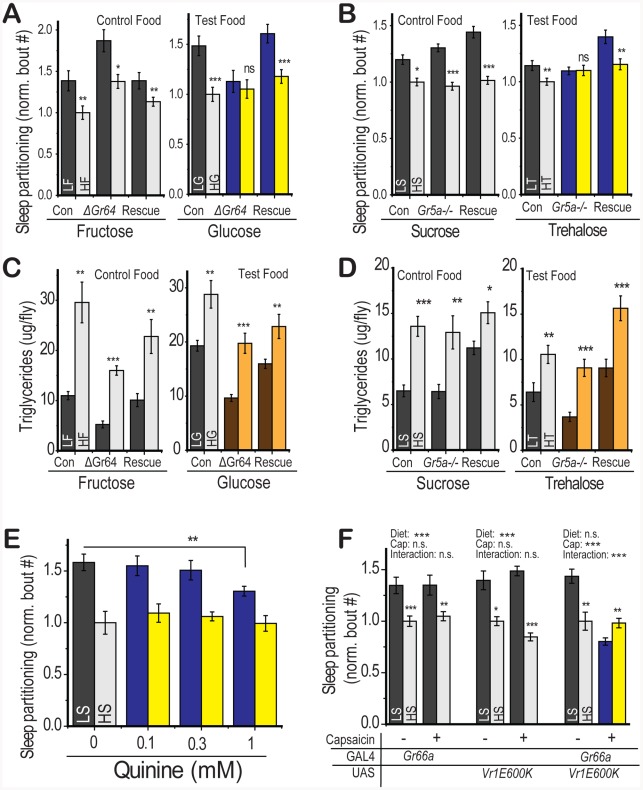
Gustatory inputs mediate sleep partitioning in response to dietary sugar. For each indicated sugar, flies were tested on a medium with 2.5% or 30% of that sugar on a 2.5% yeast base. (A) Flies with a deletion in the *Gr64a-f* sweet-sensing cluster retained sensitivity to fructose (control food) but were insensitive to a glucose-containing food (test food). This effect was rescued by expression of a UAS-*Gr64abcd_GFP_f* construct with a *Gr5a*-GAL4 promoter in the *Gr64* deletion background. (B) Flies with a deletion in the *Gr5a* trehalose receptor retained sensitivity to sucrose (control food) but were insensitive to a trehalose-containing food (test food). This effect was rescued by expression of a UAS-*Gr5a* with a *Gr5a*-GAL4 promoter. Deletion of (C) the *Gr64* cluster or (D) *Gr5a* did not compromise the rapid shift in energy storage following dietary switch on either the control or test food. (E) Quinine supplementation of the food at the indicated concentrations (significance value from one-way ANOVA followed by Fisher LSD within LS group) attenuated the sleep response to LS diet in *yw* control flies. (F) Genetic stimulation of *Gr66a*-containing neurons using the *Vr1E600K* capsaicin receptor in the presence of capsaicin was sufficient to suppress the LS-induced sleep response (significant diet∶capsaicin interaction by two-way ANOVA). There was no effect of capsaicin feeding or diet∶capsaicin interaction in control flies containing either the Gr66a-GAL4 or UAS-Vr1E600K alone but these controls retained a significant response to diet. Error bars represent mean +/− SEM for each group. *** p<0.001, ** p<0.01,* p<0.05 for all statistical calculations. Significance values for t-tests between dietary conditions are shown above each set of bars and significance values for two-way ANOVA are shown above the graph, where applicable. See Figure S4 for additional supporting evidence.

We tested whether the gustatory perception of sugar through the known gustatory sugar receptors was required for sleep partitioning. The specificity of each receptor for subsets of sugars allowed us to compare a test food that signals through that receptor and a control food of similar nutrient quality that should be unaffected by receptor manipulation. Genetic deletion of *Gr64a-f* [26] abolished the sleep response to dietary glucose (test food) and the response was rescued by expression of a UAS-*Gr64_abcd_GFP_f* construct under control of a *Gr5a*-GAL4 driver (Figure 5a). The sleep response to fructose (control food) persisted across conditions (Figure 5a), eliminating the possibility that nonspecific effects of receptor deletion on neuronal function may be blocking the diet response. We observed a partial suppression of diet-induced sleep partitioning in the *Gr64* deletion mutant when sucrose was used as the test food (Figure S4a), consistent with previous reports of partial reduction in sucrose–dependent effects on neuron firing rates following *Gr64* deletion [26], [27]. Similarly, genetic deletion of the *Gr5a* trehalose receptor [28] abolished the sleep response to dietary trehalose (test food) and the response was rescued by expression of a UAS-*Gr5a* construct under control of a *Gr5a*-GAL4 promoter (Figure 5b). The response to dietary sucrose (control food) persisted regardless of *Gr5a* genotype (Figure 5b). These results support the conclusion that activation of *Drosophila* sweet-sensing neurons through stimulation of GR5a or GR64 receptors is required for diet-induced sleep partitioning under low nutrient conditions. These findings are the first to demonstrate that sweet sensory neurons participate in the regulation of a behavioral output that is not directly associated with the ingestion of food.

We next tested whether gustatory perception of sugar was required for changes in energy storage. Diet-induced triglyceride accumulation remained intact following *Gr5a* and *Gr64* loss (Figure 5c, 5d) on both the control and test food, indicating that gustatory pathways regulate the diet-induced sleep partitioning response while sparing other pathways such as energy storage. There was no consistent relationship between total protein levels in the fly and loss of the *Gr64* or *Gr5a* locus (Figure S4b, S4c). This result further supports our finding that diet-induced changes in sleep architecture do not depend on triglyceride levels and demonstrates that deficiencies in gustatory perception do not alter all responses to dietary shift.

Appetitive responses to sugar are counteracted by stimulation of a distinct subset of taste neurons that express *Gr66a* and respond to bitter compounds [29]. Therefore we would expect that bitter compounds should suppress the sleep response to dietary sugar. Consistent with this hypothesis, stimulation of bitter-sensing neurons by dietary quinine (Figure 5e) [29] or genetic stimulation via targeted expression of the neurostimulating vanilloid receptor variant *Vr1E600K* [29] (Figure 5f) blocked the effects of dietary sugar on sleep partitioning. We note that there was no indication of sleep loss, as would be expected if the flies were simply not eating the dietary medium, in the presence of bitter stimulation (Figure S4d). We suspect that adult *Drosophila*, similar to larvae [30], can overcome gustatory-mediated food avoidance when the alternative is starvation. These results show that multiple gustatory inputs integrate to regulate sleep behavior, with appetitive inputs serving a stimulatory function and aversive inputs counteracting the stimulation. Our observations are reminiscent of recent evidence that sweet and bitter inputs oppose each other to elicit a rapid proboscis extension response in immobilized flies [31] and indicate that this simple gustatory circuit functions in a similar manner to control complex behavioral outputs.

### Diet-Induced Sleep Partitioning Is Independent of Olfactory Perception

**Figure 6:**
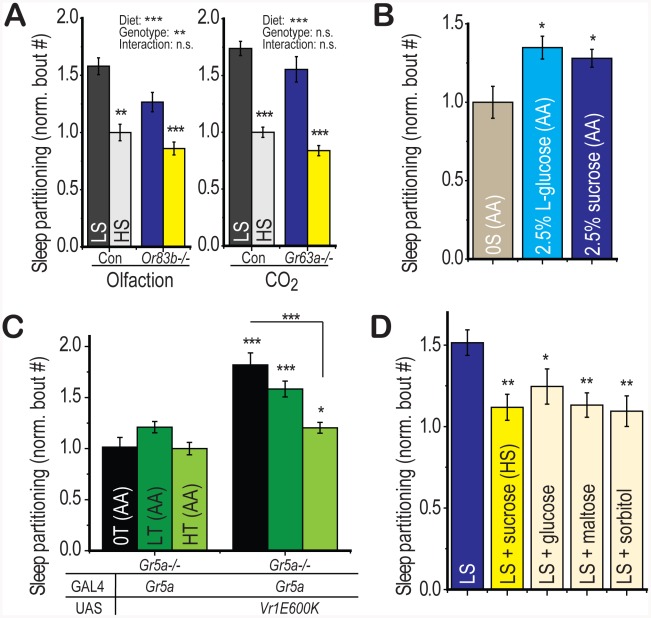
Appetitive gustatory stimulation is necessary and sufficient for diet-induced sleep partitioning. (A) Deletion of either the *Or83b* olfactory co-receptor or the *Gr63a* CO_2_ sensor is insufficient to suppress the sleep response to dietary sugar relative to genotype-matched controls (no significant diet∶genotype interaction by two-way ANOVA). (B) Activation of gustatory perception using the non-nutritional L-isomer of glucose (2.5%) in a sugar-free amino acid base food (AA+L-glucose) was sufficient to induce significant sleep partitioning relative to the amino acid base food without added sugar (0S(AA)). 2.5% sucrose in the amino acid base food (AA+sucrose) was used as a positive control. (C) Sleep partitioning was activated by ectopic expression of Vr1E600K in *Gr5a*-expressing neurons of the trehalose receptor-deficient *Gr5a−/−* mutant. Addition of dietary trehalose to a sugar-free amino acid base food (0S(AA) = 0% trehalose, LT(AA) = 2.5% trehalose, HT(AA) = 30% trehalose) was sufficient to suppress sleep partitioning in the absence of gustatory trehalose perception. (D) Addition of the non-sweet nutritional sugar alcohol sorbitol to LS food (30% total sugar) was sufficient to suppress sleep partitioning with a comparable magnitude to supplementation with the positive control sugars sucrose, glucose, and maltose. Error bars represent mean +/− SEM for each group. *** = p<0.001, ** = p<0.01,* = p<0.05 for all statistical calculations. Significance values for t-tests between dietary conditions are shown above each set of bars and significance values for two-way ANOVA are shown above the graph, where applicable. Signficance values for panels A and D are from one-way ANOVA with Fisher LSD. See also Figure S5 for additional supporting evidence and a proposed model.

Given the role for olfactory perception in the response to food sources in both *Drosophila* and *C. elegans*, we wondered whether olfactory cues could also contribute to the regulation of sleep behavior by dietary sugar. Genetic deletion of the *Or83b* olfactory co-receptor renders *Drosophila* largely anosmic [32], extends lifespan, and promotes triglyceride accumulation [33]. However, we find no evidence for an interaction between the effects of *Or83b* deletion and the effects of diet on sleep behavior (Figure 6a, left panel, two-way ANOVA p = 1×10^−7^ for diet, p = 5×10^−3^ for genotype, p = 0.3 for interaction). Deletion of the *Or83b*-independent CO_2_ receptor *Gr63a* [34] also extends lifespan and promotes triglyceride accumulation [35] but does not alter the diet-induced sleep partitioning (Figure 6a, right panel, two-way ANOVA p = 2×10^−10^ for diet, p = 0.09 for genotype, p = 0.9 for interaction). These findings together highlight the specificity of gustatory perception for the regulation of sugar-dependent behavioral responses in *Drosophila*.

### Interaction of Gustatory and Metabolic Signals

We next wondered whether stimulation of appetitive circuits would be sufficient to partition sleep behavior. The L-isomer of glucose is sufficient to activate appetitive gustatory perception in *Drosophila* but is non-nutritional [36], [37]. We found that addition of 2.5% L-glucose to the sugar-free amino acid base medium was sufficient to induce sleep partitioning relative to the base medium alone with a similar magnitude to a 2.5% sucrose positive control group (Figure 6b). Thus, sweet gustatory inputs are both necessary and sufficient to initiate the re-patterning of sleep architecture in *Drosophila*.

We have shown that the LS diet-induced sleep response is suppressed upon further addition of sugar (Figure 2c), data that support a model where dietary sugar acts as an initiator of sleep partitioning at low concentrations and a suppressor at high concentrations. We next wished to test whether this HS-induced suppression also relied on gustatory inputs. First, using flies deficient in gustatory perception of trehalose (*Gr5a−/−*), we activated gustatory neurons using *Gr5a*-GAL4 with the VR1E600K activating channel and added either zero, low, or high trehalose to the sugar-free amino acid base medium. Any suppression of sleep partitioning observed in this paradigm would be independent of GR5a-mediated trehalose perception. We observed a progressive suppression of sleep partitioning with increasing dietary trehalose (Figure 6c), indicating that while low levels of sugar promote sleep partitioning through gustatory perception, high levels suppress this function through a gustatory-independent mechanism. We further confirmed this result using sorbitol, a sweet alcohol that provides no detectable gustatory activation (Figure S5a) but is nutritionally active [37], [38]. We found that addition of sorbitol to LS food is capable of inducing suppression of the sleep partitioning response, with a similar magnitude as supplementation with the positive control sugars sucrose, glucose, and maltose (Figure 6d). Thus, we conclude that the gustatory quality of the sugar source determines the response to low concentrations of sugar but the nutritional value mediates the response to higher concentrations.

## Discussion

In this work, we have defined the effects of dietary sugar on sleep behavior. Based on these results, we propose a two-component model whereby sensory and non-sensory cues interact in an opposing manner to re-pattern sleep behavior and arousal threshold (Figure S5b). First, gustatory sensation, specifically sweet perception, serves as an initiator to stimulate sleep partitioning above the baseline level during both the day and night. Removal of appetitive gustatory inputs or competition by bitter inputs prevents this increase. Second, an additional factor independent of gustatory inputs is evoked under conditions of high dietary sugar that suppresses gustatory-mediated sleep partitioning back to baseline levels. Partial activation of this suppressor is observed even under low sugar conditions when baseline sleep partitioning has been elevated by ectopic activation of gustatory neurons. We conclude that both the gustatory and nutritional value of dietary inputs are evaluated by the organism in order to evoke complex behavioral changes. This is the first demonstration of a direct role for gustatory receptors in a behavioral phenotype not associated with feeding and these findings fit well with recent studies implicating gustatory-metabolic interplay in the regulation of appetitive memory [38] and feeding preference [39] in *Drosophila*.

We find it surprising that sleep behavior in *Drosophila* is mediated by the specific dietary addition of sugar and not total yeast content or total caloric content. However, other responses to dietary quality including fecundity [18], memory formation [40], and energy storage [4] depend on specific factors in the environmental medium. We propose that dietary sugar may provide a specific cue in the natural environment that effectively indicates identification of the periphery of a food source or the presence of nearby food sources containing higher nutrient content. Under complete starvation conditions, it is beneficial to the organism to enact an energetically costly foraging response. However, once a food source is located, selection may favor the induction of a less costly behavioral response, such as the modulation of arousal threshold, which would both induce sleep partitioning and increase responsiveness to cues associated with nearby food sources containing richer dietary content while sparing the organism a substantial energetic cost. Thus we propose that the modulation of sleep behavior by dietary quality may represent a “thrifty” alternative to other high-cost search strategies when the goal is simply to optimize nutrient acquisition rather than avoid starvation.

We find parallels between the diet-induced regulation of sleep behavior in *Drosophila* and the regulation of satiety states in other organisms. Both *Drosophila* sleep and *C. elegans* quiescence are inactive states that vary in duration based on the quality of food ingested [9]. In both systems, sensory perception and metabolic cues interact to regulate the duration of the inactive state [41]. Similarly, in mammals, the postfeeding satiety sequence results in immobile resting behavior in a manner that is modulated by the presence of bitter substances [10]. Furthermore, the orexin neuropeptides regulate sleep architecture as well as feeding/satiety behaviors in mammalian systems [11], strengthening the connection between sleep behavior and the response to dietary intake. While there is no direct homolog of the mammalian orexins in *Drosophila*, feeding behavior can be regulated by the neuropeptide NPF and the biogenic amines through mechanisms that are likely conserved. Simple modulation of these pathways substantially modulates locomotor activity and thus does not directly phenocopy the effects of dietary shift [42], [43], [44], however it is likely that the effects of diet on sleep partitioning are mediated through effects on a subset of the neurons involved in these circuits.

We find that in *Drosophila*, diet-induced sleep partitioning persists across the whole 24-hour circadian period, even though feeding is strongly regulated by circadian rhythm and primarily concentrated during the morning activity period [45]. This indicates that *Drosophila* may maintain memory of the quality of the local food source as a persistent change in arousal threshold even during portions of the circadian period when feeding is minimal. We also note that while gustatory activation occurs within minutes, sleep partitioning increases over the first several days on a low-sugar food. This result supports the hypothesis that intermittent gustatory activation during feeding periods may be inducing longer-lasting downstream changes to maintain a constant state of heightened arousal threshold while in the presence of a low-sugar food source. These changes may be mediated through the mushroom body or through changes in synaptic strength at other sites that regulate sleep behavior. A persistent change in arousal threshold and therefore sleep partitioning across the full circadian period would benefit the organism by providing increased acuity to environmental cues throughout the entire circadian period that may signal the presence of preferable food sources, conspecifics, and potential mates.

The present work adds an important piece to our understanding of the interaction between sensory perception and sleep behavior. Several lines of evidence point to a potential interaction. Both gustatory and olfactory acuity undergo circadian cycles [46], [47] and microarray analysis of the *insomniac* short-sleeping fly strains has indicated substantial differential expression in sensory perception genes [48]. However, mutants defective in the olfactory coreceptor OR83b or the C0_2_ receptor GR63a do not show a defect in total sleep regulation ([35] and personal observations). The present study indicates that sensory perception, specifically gustatory perception, is required for the transmission of information from the dietary environment to specifically regulate sleep architecture without disrupting total sleep. A similar modulatory role for sensory perception was observed by Ganguly-Fitzgerald et al. in the relationship between social interaction and sleep behavior [14]. These data support a growing role for sensory perception as an important mechanism to transmit information from the external environment to the sleep regulatory system.

It is intriguing to speculate on a possible role of diet-induced changes in sleep behavior on the suite of organism-wide responses that confer health benefits of low dietary intake. It has been proposed that the evolutionarily-conserved regulation of lifespan by dietary restriction may relate to the mechanisms of hunger and satiety. In fact, the *eat-2* mutant in *C. elegans* is both long-lived and unable to achieve a prolonged satiety-associated quiescence state, supporting a correlation between satiety behavior and longevity [9]. Changes in sleep architecture have been previously observed in association with sleep loss and occur in response to starvation [49], oxidative stress [50], aging [50], ion channel manipulations (e.g. *Sh^mns^*, *Hyperkinetic*, and *Sleepless*) [20], [51], [52], and manipulation of the biogenic amines dopamine and octopamine [21], [43]. While both stress and ion channel manipulations are associated with shortened lifespan [20], [53], manipulation of dopamine is not deleterious to longevity [54]. It is not clear whether the changes in sleep architecture contribute to a deleterious phenotype, represent a protective adaptation, or are behavioral adjustments independent of health status. However, the present work identifies sleep partitioning as a phenotype that is separable from sleep loss and associated with a longevity-inducing intervention and indicates that arousal threshold can be specifically targeted for interventions in mammalian systems.

We note that despite a very wide variation in dietary sucrose levels used in this study, most parameters remained normal, indicating that the flies show little evidence of sickness over the course of the study. Although high dietary sugar decreased overall lifespan, the only short-term changes we observed were increased triglycerides and the sleep partitioning phenotype described in this paper. Other previous reports indicate that overall fecundity is also minimally affected by changes in dietary sucrose [4]. This situation is similar to mice, where a high (50%) sucrose diet leads to obesity but minimal insulin resistance [55], and somewhat different from larval *Drosophila* where high sucrose feeding promotes rapid insulin resistance [56].

While the effects of diet on *Drosophila* do not involve sleep loss, we do predict that a reduction in the threshold for waking would lead to insomnia in human populations where the availability of sleep time is primarily restricted to the night. A recent study has found correlations between low dietary nutrient intake and the appearance of insomnia-like symptoms including shortened sleep duration [57], as would be predicted from our results. Further work will be required to investigate the potential role for dietary sugar and gustatory perception in the regulation of insomnia. We know little about the mechanisms by which sleep architecture is regulated and untangling the molecular regulatory events is likely to provide important insight into the full suite of responses to dietary quality and a deeper understanding of sleep regulation. The current model provides a plausible mechanism for the effects of dietary nutrients on sleep behavior in a model organism, which may illuminate conserved mechanisms that metazoans use to detect environmental change and respond appropriately.

## Materials and Methods

### Fly Strains and Husbandry

Flies were a kind gift from the indicated laboratories: *Per^01^*, *Tim^01^*, *Pdf^01^*, *Cyc^01^* (P. Hardin); *Or83b* ^−/−^ (L. Vosshall [32]); *Gr5a*-GAL4, UAS-*Gr5a* (A. Dahanukar [28]); *Lsd-2^51^*, *Lsd-2^rev^* (R. Kuhnlein [58]); .Δ*Gr64*, control, UAS-*Gr64abcd_GFP_f*, *Gr66a*-GAL4 (H. Amrein, [26]); *Gr5aΔ5*, and *Gr63a* ^−/−^ (J. Carlson, [28], [34]); UAS-*VR1E600K* (K. Scott, [29]). *Sh^mns^* flies were obtained from the Bloomington *Drosophila* Stock Center. Flies were compared to control strains from at least 6–8 generations of backcrossing in our laboratory.

All fly lines used for experiments were placed into environment controlled chambers on a 12∶12 hour light dark cycle and mated for 24 h on grape agar plates to achieve a large synchronized population of flies for intercomparison. Eggs were distributed at a controlled density onto cornmeal-agar food for larval development. Adult flies were collected within 24 hours of eclosion, mated for 2 days on 10∶10% sucrose∶yeast food and then separated by sex and maintained at a constant density until experimentation. We used food prepared as described previously [4]. Briefly, sucrose and yeast were added as specified to 1.5% agar, cooked for approximately 1 hour, and then cooled with stirring and distributed into vials. Where indicated, RU486 (Sigma, 200 uM), quinine (Sigma, indicated concentrations), and capsaicin (Sigma, 100 uM) were added to food immediately before pouring into vials, between 55 and 60°C.

### Behavioral Monitoring

We used the DAMS activity monitoring system (Trikinetics Waltham, MA) for recording photobeam crosses by individual flies using 65 mm×5 mm polycarbonate tubes. Summary and statistical analyses were conducted using custom scripts for the R statistical computing platform [59]. To avoid confounding effects of female egg-laying behavior on sleep behavior characteristics, young males (between 8 and 20 days) were used except where indicated. At least 16 flies were used per group, and monitor locations were randomized within an experiment to avoid position effects. Data were collected in 1 minute bins for 5–6 days, and the first day was discarded to avoid effects of CO_2_ anesthesia and adjustment to the monitor tubes. Sleep was defined as periods of at least 5 consecutive minutes of inactivity [12], [13]. Sleep bouts were defined as the number of uninterrupted periods of 5 or more minutes of inactivity. The number of sleep bouts was normalized to the mean value of the control group within each experimental cohort and presented as the sleep partitioning score (normalized bout number). Rest was defined as periods of inactivity totaling less than 5 minutes in duration. Sleep latency was defined as the time between a lighting transition and the first sleep bout. All test conditions were compared directly to contemporaneous controls to ensure a consistent larval growth period and consistent activity monitoring conditions.

For the video monitoring assays, video cameras (miniature box-format 1/3″ Sony Super HAD CCD with a vari-focal lens and 0.5 lux sensitivity) were suspended ∼25 cm above 65 mm×5 mm polycarbonate tubes containing food at one end and cotton at the other. The tubes were mounted in white polyethylene (8 tubes/block) to provide maximum contrast while preventing visual detection between flies in nearby tubes. Activity was recorded across the entire 12-hour circadian light phase for three consecutive days. We used the VideoFly analysis software (developed in our laboratory) to track movements of individual flies relative to a composite background image. Each image was first subtracted from a background composite and adjusted to maximize signal-to-noise ratio. All image adjustment steps were applied identically to all movies. The X and Y position of the centroid for each spot (fly) was calculated for each frame. We performed quality control analysis on each movie to ensure that all flies were detected in each frame. Movement was evaluated as the distance between centroid positions for 2 consecutive frames. The present analysis was conducted at 1 frame per second for 15 flies/group. Sleep was defined as periods where the centroid position moved less than 2 pixels for a minimum of 5 minutes. A feeding bout consisted of a continuous period where the centroid position was within 1 body length of the food.

Except where indicated, experiments were conducted in 12∶12 hour cycles of light∶dark in an environment-controlled incubator. Light, temperature, and humidity were maintained at 60–2500 lux, 25°C, and 60% respectively.

For the two-choice food preference assay we followed the protocol of Dus et al. for gustatory-dependent preference [39]. Briefly, 10 groups of 5 flies were switched to agar for 5 hours prior to the assay period. Flies were then placed in 60-well plates containing an equal number of wells with the test sugar (as indicated) and agar containing either FD&C Blue #1 (0.05%) or FD&C Red #3 (0.05%). A single trial consisted of 2 plates with the dye colors switched relative to the food to avoid confounds associated with potential dye preference. Five trials were performed per condition.

### Sleep Deprivation

Trikenetics DAM2 monitors were mounted on a vortex shaking device following 2 days of acclimatization. During the deprivation period, a shaking stimulus was delivered for 2 seconds every minute, with the stimulus applied at a random location within that minute in order to reduce acclimitization. This protocol resulted in >90% sleep loss during the deprivation period, as previously reported [52].

### Metabolic Assays

Flies were exposed to the test treatment either in individual monitoring tubes for 6 days. At the end of the treatment, the flies were frozen at −20°C and homogenized in 50 ul/fly of cold PBS+0.05% Triton X-100 using a TissueLyser bead mill (Qiagen). The homogenate was filtered to remove large particles. Triglyceride and glucose levels were measured on randomized samples in 96 well plate format using the Infinity™ reagent system (Roche). Glycogen was measured by first converting glycogen to glucose using 0.1 mU amyloglucosidase/20 ul buffer at 37 degrees for 30 minutes and then measuring released glucose. Protein was measured using the bicinchoninic acid (BCA) method (Pierce). Each column represents the data from at least 16 flies per group. Trehalose was measured according to Chen et al. [60]. Briefly, each fly was homogenized in 50 ul 0.25 M NaCO_3_ using the bead mill and incubated at 95°C for 2 hours. 30 ul of 1 M Acetic Acid and 120 ul of 0.25 M Na-Acetate were added and incubated overnight at 37°C with 0.05 U/ml Porcine Kidney Trehalase (Sigma) to liberate glucose or with vehicle control. Glucose concentrations were measured using the Infinity™ Glucose reagent.

### Statistical Analysis

P-values are noted in the figures as * p<0.05,** p<0.01,*** p<0.001. We used Welch's t-test for pairwise comparisons. Multivariate ANOVA was used for inference about the effects of each of 2 variables and their interaction on the experimental outcome. ANOVA results are summarized above each plot, where applicable. Other tests are noted in the text. All calculations were performed using the R statistical software package.

## Supporting Information

Figure S1 Additional support for Figure 1. (A) Total sleep was calculated for each point in the day using a 30 minute moving average. Shown is the mean +/− SEM for 16 male control (*yw*) flies on low (5∶5% sucrose∶yeast) and high 20∶20% sucrose∶yeast) nutrient food for a full 24-hour day under 12∶12 hour light∶dark conditions. (B) Canton-S (left) and *yw* (right) male flies were assayed for longevity in the DAMS activity tube environment using the food conditions assayed in the subsequent experiments. LS = 2.5% sucrose∶yeast and HS = 30% sucrose, 2.5% yeast. Flies were placed in individual tubes beginning on adult day 2 and food was changed every 5 days throughout the lifespan. Time of death was recorded as the time at which there were no activity counts for a period of 12 hours. P-values are derived from the Log-rank test. (C) Food preparation method and early-life housing density are environmental factors that impact the baseline sleep architecture but there was no significant interaction with the effects of diet. (D) The normalized sleep partitioning score was compared to the total number of sleep bouts across multiple control experiments. There was no significant trend between the normalized values and the underlying number of total sleep bouts. (E) Specialized activity tubes containing small air holes along the length of the tube were used in order to test the effects of dietary sugar on sleep partitioning in the presence of an alternate water source. In this paradigm, food (LS = 2.5% sucrose and HS = 30% sucrose in a 2.5% yeast base food) was provided at one end of the tube and 0.5% agar was provided at the other end. (F) The effects of dietary sucrose concentration (LS = 2.5% sucrose and HS = 30% sucrose) on sleep partitioning were tested in a standard sucrose-agar medium without yeast that is commonly used for *Drosophila* behavioral analysis. Error bars represent mean +/− SEM for each group. *** = p<0.001, ** = p<0.01,* = p<0.05 for all statistical calculations. Error bars represent mean +/− SEM for each group. *** p<0.001, ** p<0.01,* p<0.05 for all statistical calculations. Significance values for t-tests between dietary conditions are shown above each set of bars and significance values for two-way ANOVA are shown above the graph, where applicable.
(PDF)

Figure S2 Additional support for Figure 2. (A) Onset of sleep response following dietary shift was progressive and sustained in the Canton-S (CS) control strain, similar to the results presented in Figure 2B for the *yw* control strain. P-values are from t-tests following one-way ANOVA. (B) The effects of dietary sugar are completely reversible. Young control (*yw*) flies were exposed to Diet 1 (indicated below the graph) and then switched after 6 days of recording to diet 2. P-values indicated are from two-way ANOVA.Flies housed on sugar-free 0S amino acid base medium do not show any signs of starvation or general sickness including (C) no significant difference between groups in overall activity (one-way ANOVA) and (D) no significant effect of time on sleep loss relative to day 1 (black symbols, one-way ANOVA). When the 0S medium was diluted 2×, we did observe progressive sleep loss (red symbols, one-way ANOVA with Fisher's LSD), indicating that the nutrient levels in the undiluted medium are sufficient to maintain health through the measurement period. Error bars represent mean +/− SEM for each group. *** p<0.001, ** p<0.01,* p<0.05 for all statistical calculations.
(PDF)

Figure S3 Additional support for Figure 3. We used the UAS-*TrpA1* temperature-sensitive ion channel under control of the *Tdc2*-GAL4 promoter to ectopically activate octopamine neurons and induce sleep loss at the test temperature. Flies were tested for 4 days at the control temperature (22°C, left panel) and then switched to the test temperature (29°C, right panel) for 4 days. P-values are from t-tests following one-way ANOVA. Error bars represent mean +/− SEM for each group. *** p<0.001, ** p<0.01,* p<0.05 for all statistical calculations.
(PDF)

Figure S4 Additional support for Figure 5. (A, left panel) We observed a partial suppression of the diet-induced sleep response in *Gr64* deletion flies using sucrose as the test food in experiments conducted simultaneous to those presented in Figure 5a. (A, right panel) *Gr64* deletion did not suppress diet-induced TAG accumulation when sucrose was used as the test food. (B,C) Protein levels from flies assayed for triglyceride response to diet in Figure 5c, 5d. (D) Average total sleep per day was analyzed for the experiments presented in Figure 5e and 5f. There was no significant change across groups by one-way ANOVA or by t-tests comparing across the presence and absence of the aversive stimulus. (D) Average total sleep per day was analyzed for the experiments presented in Figure 5e and 5f. There was no significant change across groups by one-way ANOVA or by t-tests comparing across the presence and absence of the aversive stimulus. Error bars represent mean +/− SEM for each group. *** p<0.001, ** p<0.01,* p<0.05 for all statistical calculations. P-values are from t-tests within each genotype.
(PDF)

Figure S5 Additional support for Figure 6 and a proposed model. (A) We measured preference behavior for 30% sorbitol relative to agar alone in the two-choice assay (5 groups of 10 flies each). An identical positive control cohort was tested in parallel with 30% sucrose and agar. (B) Model: We have determined that the presence of dietary sugar promotes sleep partitioning through activation of sweet (Gr64 and Gr5a-dependent) gustatory perception. Elevated sleep partitioning is coupled with, and likely caused by, a sustained increase in the probability of arousal from sleep. This change in arousal threshold would thereby increase responsiveness to cues from nearby nutrient-rich food sources. Activation of bitter (Gr66a-dependent) gustatory neurons blocks the effects of sweet perception on sleep behavior. Upon further addition of sugar, a non-gustatory suppressor is activated that counteracts the effects of gustatory stimulation on sleep behavior. Diet-induced triglyceride accumulation is also mediated by a gustatory-independent pathway. We propose that the modulation of sleep behavior (specifically arousal threshold) by low dietary sugar as a novel mechanism that would support the identification of alternative feeding sites with richer nutritional content without enacting an energetically costly foraging response.
(PDF)

Table S1 Changes in activity and sleep parameters in response to modulation of dietary yeast and sugar. Sleep is defined as periods of 5 or more minutes of inactivity. Rest is defined as periods of 1–4 consecutive minutes of inactivity. Brief awakening is defined as a wake period of only one minute duration. *** p<0.001, ** p<0.01,* p<0.05 for the comparison between diets (t-test).
(PDF)

Table S2 Activity and sleep parameters for three control genotypes in response to dietary sugar. Sleep is defined as periods of 5 or more minutes of inactivity. Rest is defined as periods of 1–4 consecutive minutes of inactivity. Brief awakening is defined as a wake period of only one minute duration. *** p<0.001, ** p<0.01,* p<0.05 for the comparison between diets (t-test).
(PDF)
