# Supplementary figures and images for: Re-Patterning Sleep Architecture in Drosophila through Gustatory Perception and Nutritional Quality
Source: PLoS Genet. 2012 May 3;8(5):e1002668. doi: 10.1371/journal.pgen.1002668 (PMC3342939; doi:10.1371/journal.pgen.1002668)

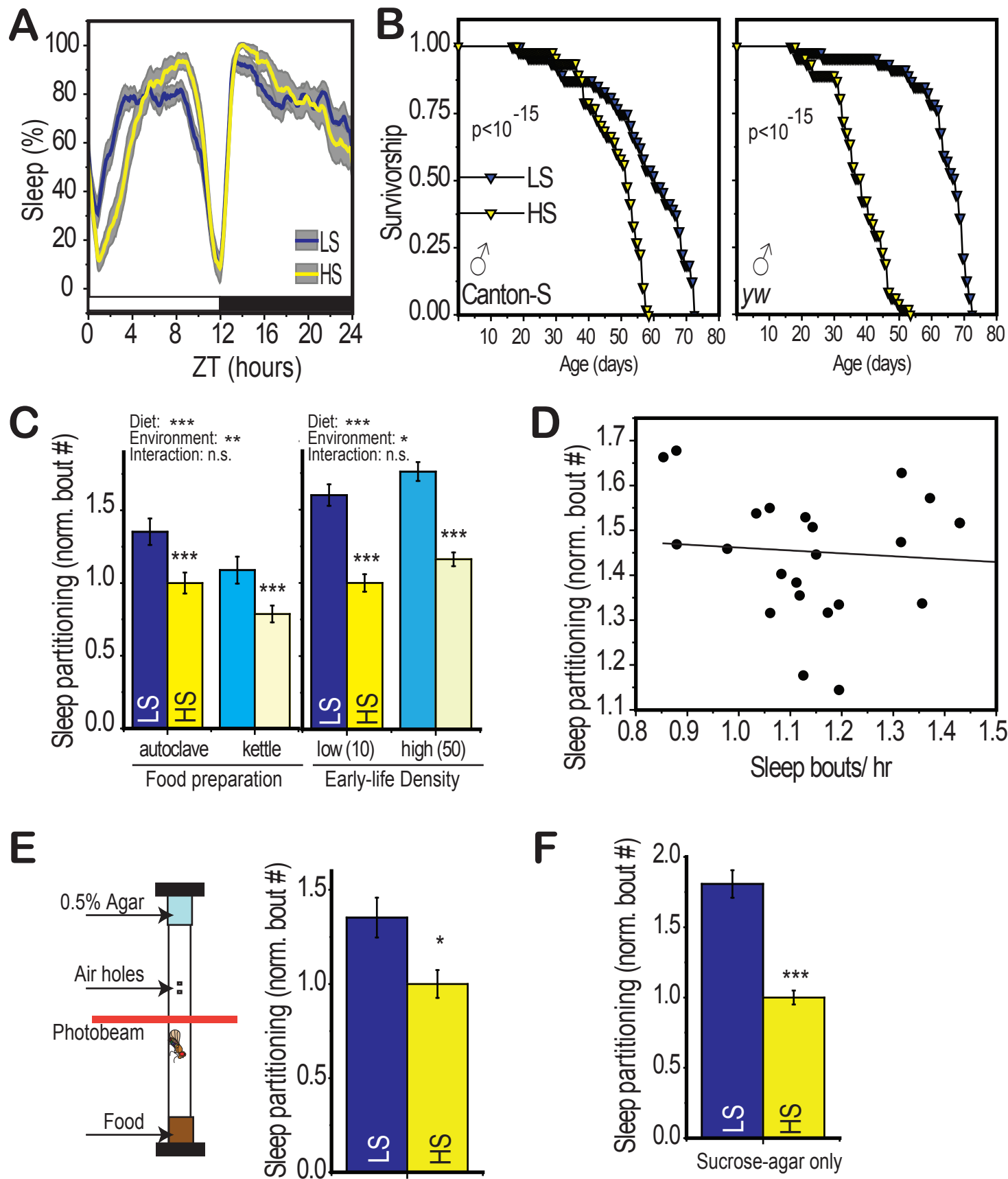

Figure S1

Supplement: Figure S1 — Additional support for Figure 1. (A) Total sleep was calculated for each point in the day using a 30 minute moving average. Shown is the mean +/− SEM for 16 male control (yw) flies on low (5∶5% sucrose∶yeast) and high 20∶20% sucrose∶yeast) nutrient food for a full 24-hour day under 12∶12 hour light∶dark conditions. (B) Canton-S (left) and yw (right) male flies were assayed for longevity in the DAMS activity tube environment using the food conditions assayed in the subsequent experiments. LS = 2.5% sucrose∶yeast and HS = 30% sucrose, 2.5% yeast. Flies were placed in individual tubes beginning on adult day 2 and food was changed every 5 days throughout the lifespan. Time of death was recorded as the time at which there were no activity counts for a period of 12 hours. P-values are derived from the Log-rank test. (C) Food preparation method and early-life housing density are environmental factors that impact the baseline sleep architecture but there was no significant interaction with the effects of diet. (D) The normalized sleep partitioning score was compared to the total number of sleep bouts across multiple control experiments. There was no significant trend between the normalized values and the underlying number of total sleep bouts. (E) Specialized activity tubes containing small air holes along the length of the tube were used in order to test the effects of dietary sugar on sleep partitioning in the presence of an alternate water source. In this paradigm, food (LS = 2.5% sucrose and HS = 30% sucrose in a 2.5% yeast base food) was provided at one end of the tube and 0.5% agar was provided at the other end. (F) The effects of dietary sucrose concentration (LS = 2.5% sucrose and HS = 30% sucrose) on sleep partitioning were tested in a standard sucrose-agar medium without yeast that is commonly used for Drosophila behavioral analysis. Error bars represent mean +/− SEM for each group. *** = p<0.001, ** = p<0.01,* = p<0.05 for all statistical calculations. Error bars [file pgen.1002668.s001.pdf]

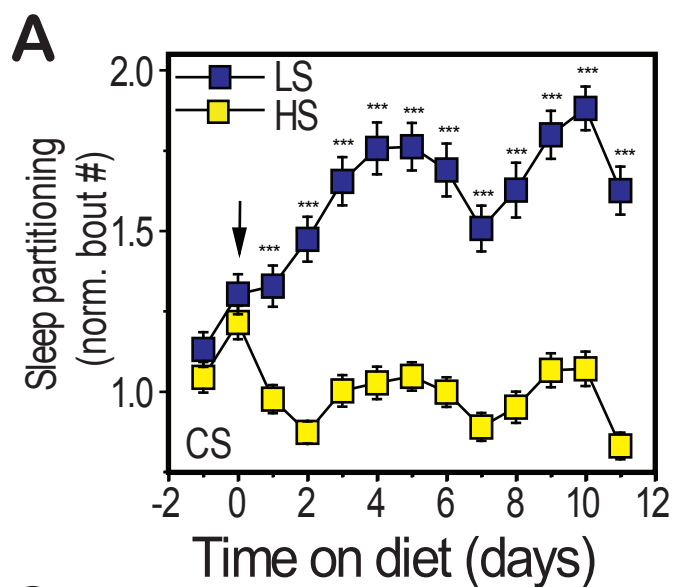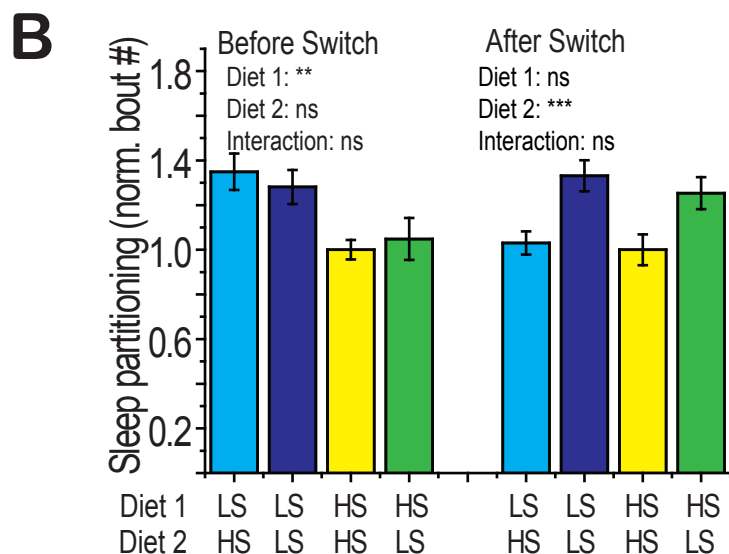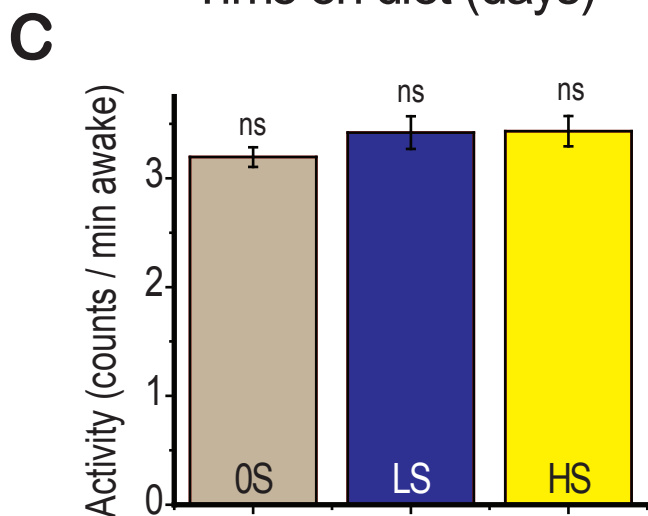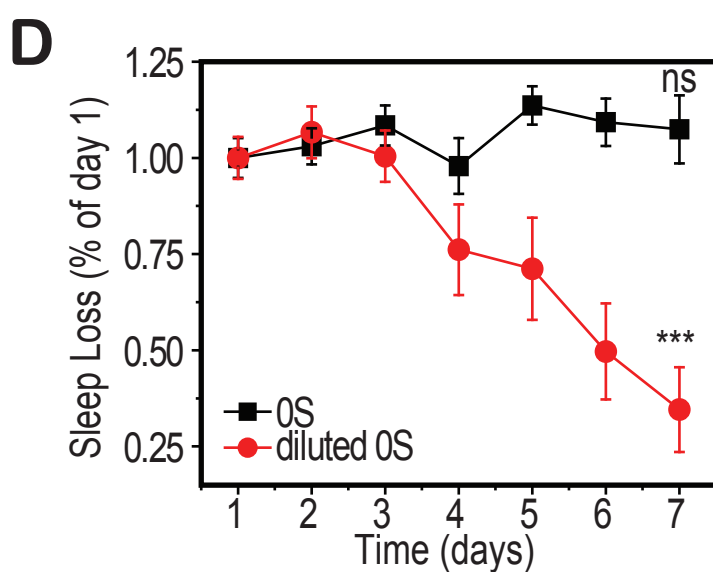

Figure S2

Supplement: Figure S2 — Additional support for Figure 2. (A) Onset of sleep response following dietary shift was progressive and sustained in the Canton-S (CS) control strain, similar to the results presented in Figure 2B for the yw control strain. P-values are from t-tests following one-way ANOVA. (B) The effects of dietary sugar are completely reversible. Young control (yw) flies were exposed to Diet 1 (indicated below the graph) and then switched after 6 days of recording to diet 2. P-values indicated are from two-way ANOVA.Flies housed on sugar-free 0S amino acid base medium do not show any signs of starvation or general sickness including (C) no significant difference between groups in overall activity (one-way ANOVA) and (D) no significant effect of time on sleep loss relative to day 1 (black symbols, one-way ANOVA). When the 0S medium was diluted 2×, we did observe progressive sleep loss (red symbols, one-way ANOVA with Fisher's LSD), indicating that the nutrient levels in the undiluted medium are sufficient to maintain health through the measurement period. Error bars represent mean +/− SEM for each group. *** p<0.001, ** p<0.01,* p<0.05 for all statistical calculations. (PDF) [file pgen.1002668.s002.pdf]

**A**

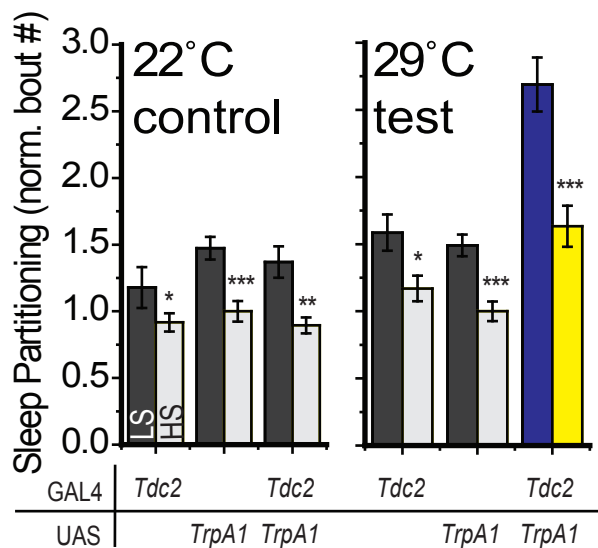

Figure S3

Supplement: Figure S3 — Additional support for Figure 3. We used the UAS-TrpA1 temperature-sensitive ion channel under control of the Tdc2-GAL4 promoter to ectopically activate octopamine neurons and induce sleep loss at the test temperature. Flies were tested for 4 days at the control temperature (22°C, left panel) and then switched to the test temperature (29°C, right panel) for 4 days. P-values are from t-tests following one-way ANOVA. Error bars represent mean +/− SEM for each group. *** p<0.001, ** p<0.01,* p<0.05 for all statistical calculations. (PDF) [file pgen.1002668.s003.pdf]

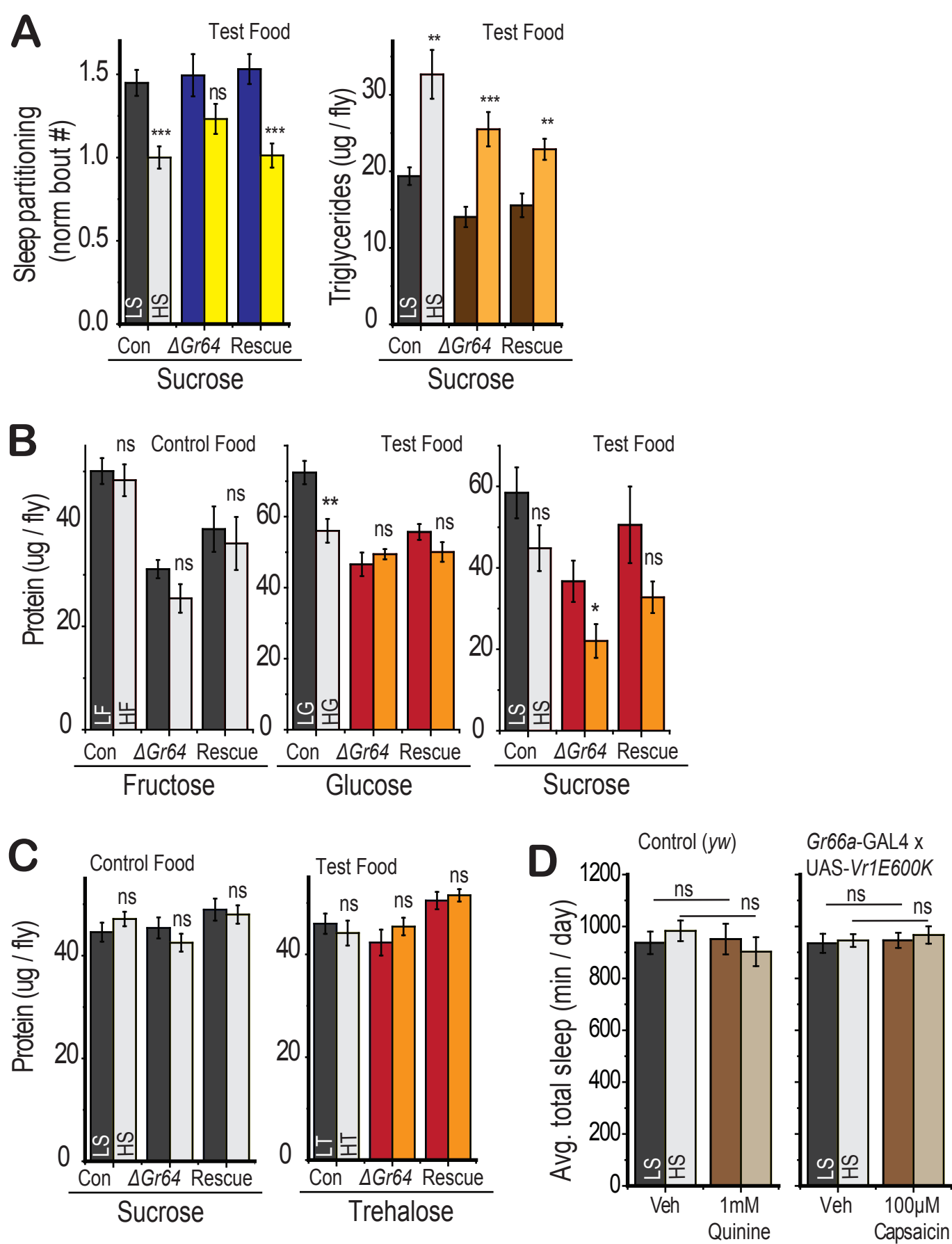

Figure S4

Supplement: Figure S4 — Additional support for Figure 5. (A, left panel) We observed a partial suppression of the diet-induced sleep response in Gr64 deletion flies using sucrose as the test food in experiments conducted simultaneous to those presented in Figure 5a. (A, right panel) Gr64 deletion did not suppress diet-induced TAG accumulation when sucrose was used as the test food. (B,C) Protein levels from flies assayed for triglyceride response to diet in Figure 5c, 5d. (D) Average total sleep per day was analyzed for the experiments presented in Figure 5e and 5f. There was no significant change across groups by one-way ANOVA or by t-tests comparing across the presence and absence of the aversive stimulus. (D) Average total sleep per day was analyzed for the experiments presented in Figure 5e and 5f. There was no significant change across groups by one-way ANOVA or by t-tests comparing across the presence and absence of the aversive stimulus. Error bars represent mean +/− SEM for each group. *** p<0.001, ** p<0.01,* p<0.05 for all statistical calculations. P-values are from t-tests within each genotype. (PDF) [file pgen.1002668.s004.pdf]

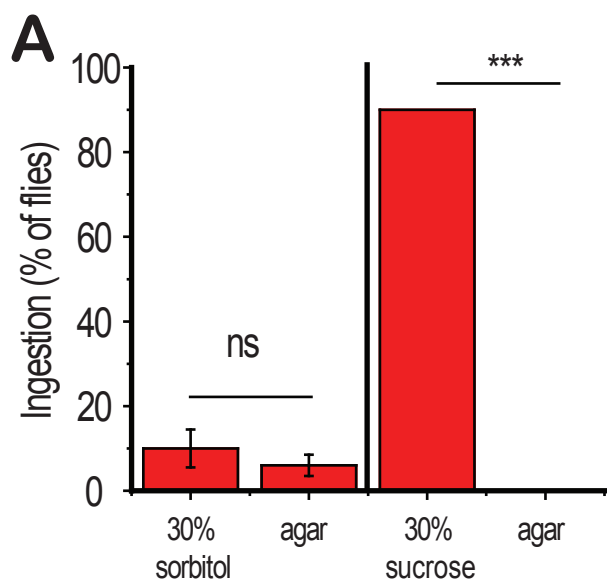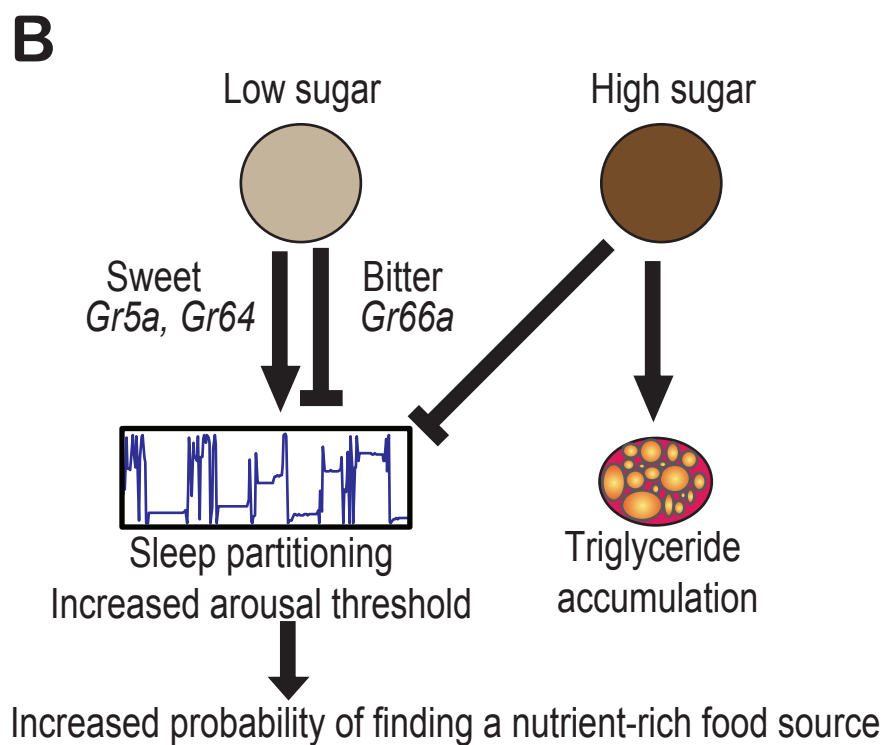

Figure S5

Supplement: Figure S5 — Additional support for Figure 6 and a proposed model. (A) We measured preference behavior for 30% sorbitol relative to agar alone in the two-choice assay (5 groups of 10 flies each). An identical positive control cohort was tested in parallel with 30% sucrose and agar. (B) Model: We have determined that the presence of dietary sugar promotes sleep partitioning through activation of sweet (Gr64 and Gr5a-dependent) gustatory perception. Elevated sleep partitioning is coupled with, and likely caused by, a sustained increase in the probability of arousal from sleep. This change in arousal threshold would thereby increase responsiveness to cues from nearby nutrient-rich food sources. Activation of bitter (Gr66a-dependent) gustatory neurons blocks the effects of sweet perception on sleep behavior. Upon further addition of sugar, a non-gustatory suppressor is activated that counteracts the effects of gustatory stimulation on sleep behavior. Diet-induced triglyceride accumulation is also mediated by a gustatory-independent pathway. We propose that the modulation of sleep behavior (specifically arousal threshold) by low dietary sugar as a novel mechanism that would support the identification of alternative feeding sites with richer nutritional content without enacting an energetically costly foraging response. (PDF) [file pgen.1002668.s005.pdf]
