# Supplementary material for: Re-Patterning Sleep Architecture in Drosophila through Gustatory Perception and Nutritional Quality
Source: PLoS Genet. 2012 May 3;8(5):e1002668. doi: 10.1371/journal.pgen.1002668 (PMC3342939; doi:10.1371/journal.pgen.1002668)
Supplement: Table S1 — Changes in activity and sleep parameters in response to modulation of dietary yeast and sugar. Sleep is defined as periods of 5 or more minutes of inactivity. Rest is defined as periods of 1–4 consecutive minutes of inactivity. Brief awakening is defined as a wake period of only one minute duration. *** p<0.001, ** p<0.01,* p<0.05 for the comparison between diets (t-test). (PDF) [file pgen.1002668.s006.pdf]

	Trial 1 (n=16/group)			Trial 2 (n=16/group)			Trial 3 (n=16/group)		
Sugar	5%	20%		5%	20%		5%	20%	
Yeast	5%	20%		5%	20%		5%	20%	
Activity ± SEM (counts/min)									
All	0.49 ± 0.05	0.61 ± 0.04		0.55 ± 0.05	0.52 ± 0.05		0.46 ± 0.04	0.41 ± 0.03	
Awake	2.62 ± 0.24	2.31 ± 0.17		**2.66 ± 0.14**	**2.15 ± 0.11**	*	2.37 ± 0.13	2.08 ± 0.09	
Night	0.44 ± 0.06	0.57 ± 0.05		0.45 ± 0.05	0.36 ± 0.06		0.39 ± 0.05	0.33 ± 0.04	
Day	0.54 ± 0.05	0.67 ± 0.04		0.65 ± 0.06	0.67 ± 0.05		0.53 ± 0.04	0.49 ± 0.04	
Sleep ± SEM (min/hr)									
All	42.0 ± 2.0	37.6 ± 3.3		42.6 ± 1.4	40.5 ± 1.6		42.3 ± 2.2	41.6 ± 1.8	
Night	43.9 ± 2.3	39.9 ± 3.1		47.2 ± 1.6	47.0 ± 2.0		45.8 ± 2.2	46.4 ± 1.8	
Day	40 ± 1.9	35.3 ± 3.6		37.9 ± 1.4	33.9 ± 1.5		38.5 ± 2.4	36.4 ± 1.9	
Sleep bouts ± SEM (events/hr)									
All	**1.86 ± 0.17**	**0.91 ± 0.16**	***	**1.64 ± 0.12**	**1.1 ± 0.08**	**	**1.54 ± 0.13**	**1.11 ± 0.08**	*
Night	**1.85 ± 0.19**	**0.92 ± 0.16**	**	**1.53 ± 0.14**	**1.04 ± 0.11**	*	**1.38 ± 0.16**	**0.97 ± 0.1**	*
Day	**1.88 ± 0.16**	**0.89 ± 0.16**	***	**1.77 ± 0.13**	**1.16 ± 0.08**	***	**1.71 ± 0.12**	**1.26 ± 0.08**	*
Rest ± SEM (min/hr)									
All	5.37 ± 0.68	5.9 ± 1.17		4.96 ± 0.44	4.95 ± 0.48		5.44 ± 0.79	5.98 ± 0.74	
Night	4.73 ± 0.82	5.03 ± 1.12		3.59 ± 0.45	3.32 ± 0.52		4.33 ± 0.77	4.04 ± 0.64	
Day	6.02 ± 0.62	6.81 ± 1.25		6.34 ± 0.56	6.61 ± 0.56		6.63 ± 0.91	8.07 ± 0.9	
Brief awakening ± SEM (events/hr)									
All	2.2 ± 0.22	1.66 ± 0.34		1.99 ± 0.13	1.68 ± 0.16		2.02 ± 0.23	1.96 ± 0.25	
Night	2.09 ± 0.26	1.49 ± 0.31		1.63 ± 0.15	1.24 ± 0.17		1.66 ± 0.22	1.34 ± 0.18	
Day	2.31 ± 0.22	1.83 ± 0.38		2.34 ± 0.19	2.12 ± 0.19		2.4 ± 0.28	2.62 ± 0.35	
Wake ± SEM (min/hr)									
All	12.6 ± 1.4	16.4 ± 2.2		12.5 ± 1.0	14.6 ± 1.3		12.3 ± 1.4	12.4 ± 1.1	
Night	11.3 ± 1.6	15.0 ± 2.0		9.2 ± 1.2	9.7 ± 1.5		9.9 ± 1.5	9.5 ± 1.2	
Day	14.0 ± 1.4	17.9 ± 2.4		**15.8 ± 1.1**	**19.5 ± 1.2**	*	14.9 ± 1.5	15.5 ± 1.2	
Mean sleep bout length ± SEM (min)									
All	26.8 ± 3.9	52.2 ± 11.5		**28.5 ± 2.6**	**39.2 ± 3.7**	*	30.8 ± 3.7	41.2 ± 3.9	
Night	30.4 ± 5.4	53.7 ± 11.1		36.5 ± 4.2	51.2 ± 7.8		38.7 ± 6.2	49.7 ± 8.1	
Day	24.2 ± 3.1	51.7 ± 12.5		**23.3 ± 2.1**	**31.3 ± 2.1**	*	**26.1 ± 3.3**	**37.6 ± 3.6**	*
Latency to first sleep following light transition ± SEM (min)									
Night	29.2 ± 6.0	59.9 ± 19.2		13.8 ± 1.9	46.5 ± 25.8		38.2 ± 7.6	66.4 ± 12.7	
Day	25.5 ± 4.3	80.2 ± 23.4		44.9 ± 9.7	69.1 ± 12.6		31.8 ± 14.8	65.2 ± 23.7	
