# Supplementary material for: Re-Patterning Sleep Architecture in Drosophila through Gustatory Perception and Nutritional Quality
Source: PLoS Genet. 2012 May 3;8(5):e1002668. doi: 10.1371/journal.pgen.1002668 (PMC3342939; doi:10.1371/journal.pgen.1002668)
Supplement: Table S2 — Activity and sleep parameters for three control genotypes in response to dietary sugar. Sleep is defined as periods of 5 or more minutes of inactivity. Rest is defined as periods of 1–4 consecutive minutes of inactivity. Brief awakening is defined as a wake period of only one minute duration. *** p<0.001, ** p<0.01,* p<0.05 for the comparison between diets (t-test). (PDF) [file pgen.1002668.s007.pdf]

	Canton-S #1 (n=16 / group)			Canton-S #2 (n=16 / group)			*yw* #1 (n=16 / group)			*yw* #2 (n=16 / group)			w^{1118} #1 (n=16 / group)			w^{1118} #2 (n=16 / group)		
Sugar	2.5%	30%		2.5%	30%		2.5%	30%		2.5%	30%		2.5%	30%		2.5%	30%	
Yeast	2.5%	2.5%		2.5%	2.5%		2.5%	2.5%		2.5%	2.5%		2.5%	2.5%		2.5%	2.5%	
Activity ± SEM (counts/min)																		
All	0.74 ± 0.06	0.92 ± 0.09		0.85 ± 0.06	0.92 ± 0.09		0.5 ± 0.04	0.54 ± 0.04		0.87 ± 0.06	0.81 ± 0.06		0.64 ± 0.04	0.71 ± 0.05		1.19 ± 0.09	1.1 ± 0.09	
Awake	2.66 ± 0.09	2.94 ± 0.13		2.75 ± 0.11	2.64 ± 0.08		2.36 ± 0.07	2.37 ± 0.1		2.76 ± 0.12	2.56 ± 0.08		**2.69 ± 0.08**	**3.15 ± 0.09**	***	**3.63 ± 0.09**	**4.07 ± 0.17**	*
Night	0.61 ± 0.05	0.79 ± 0.11		0.71 ± 0.07	0.87 ± 0.1		0.43 ± 0.05	0.43 ± 0.05		0.67 ± 0.07	0.57 ± 0.04		0.6 ± 0.04	0.66 ± 0.05		1.03 ± 0.12	0.74 ± 0.08	
Day	0.87 ± 0.07	1.04 ± 0.09		0.99 ± 0.08	0.98 ± 0.09		0.57 ± 0.04	0.65 ± 0.05		1.08 ± 0.09	1.04 ± 0.11		0.68 ± 0.04	0.77 ± 0.05		1.37 ± 0.08	1.51 ± 0.11	
Sleep ± SEM (min/hr)																		
All	37.8 ± 1.4	36.7 ± 1.6		36.4 ± 1.5	34.2 ± 1.5		40.7 ± 1.3	39.6 ± 1.7		31.2 ± 1.9	32.4 ± 1.9		42.1 ± 0.8	44.1 ± 1.0		**35.6 ± 1.4**	**40.5 ± 1.3**	*
Night	43.2 ± 1.6	42.5 ± 1.8		43.2 ± 1.6	40.1 ± 2.1		45.2 ± 1.5	45.3 ± 1.6		38.6 ± 2.1	40.1 ± 1.9		44.9 ± 0.9	46.7 ± 0.8		**38.2 ± 2.0**	**45.0 ± 1.6**	*
Day	32.5 ± 1.7	31.1 ± 2.3		29.3 ± 1.7	28.1 ± 1.5		36.1 ± 1.5	33.8 ± 2.1		23.8 ± 2.4	24.7 ± 3.4		39.0 ± 0.9	41.2 ± 1.3		32.6 ± 1.1	35.4 ± 1.3	
Sleep bouts ± SEM (events/hr)																		
All	**1.38 ± 0.08**	**1.13 ± 0.06**	*	**1.44 ± 0.09**	**1.05 ± 0.09**	**	**1.59 ± 0.1**	**1.19 ± 0.09**	**	**1.81 ± 0.08**	**1.36 ± 0.1**	**	**2.17 ± 0.08**	**1.43 ± 0.06**	***	**1.73 ± 0.08**	**1.45 ± 0.07**	*
Night	**1.47 ± 0.13**	**1.01 ± 0.07**	**	**1.27 ± 0.11**	**0.8 ± 0.1**	**	1.42 ± 0.13	1.19 ± 0.1		**1.5 ± 0.09**	**1.05 ± 0.12**	**	**1.91 ± 0.12**	**1.17 ± 0.06**	***	**1.55 ± 0.13**	**1.14 ± 0.08**	*
Day	1.3 ± 0.07	1.25 ± 0.11		**1.61 ± 0.1**	**1.3 ± 0.1**	**	**1.77 ± 0.11**	**1.2 ± 0.1**	***	**2.13 ± 0.13**	**1.67 ± 0.14**	*	**2.45 ± 0.05**	**1.71 ± 0.08**	***	1.94 ± 0.08	1.79 ± 0.09	
Rest ± SEM (min/hr)																		
All	5.14 ± 0.35	4.38 ± 0.47		4.8 ± 0.34	5.21 ± 0.39		6.23 ± 0.58	6.14 ± 0.64		8.83 ± 0.64	8.22 ± 0.76		**3.42 ± 0.31**	**2.17 ± 0.2**	**	**4.38 ± 0.38**	**3.05 ± 0.34**	*
Night	**4.23 ± 0.46**	**2.74 ± 0.37**	*	2.79 ± 0.27	2.93 ± 0.45		4.45 ± 0.52	4.23 ± 0.47		5.49 ± 0.64	5.59 ± 0.73		**2.47 ± 0.29**	**1.53 ± 0.16**	*	**3.57 ± 0.5**	**2.16 ± 0.39**	*
Day	6.03 ± 0.38	5.97 ± 0.66		6.88 ± 0.49	7.58 ± 0.43		8.05 ± 0.77	8.1 ± 0.9		12.2 ± 0.83	10.89 ± 1.35		**4.48 ± 0.44**	**2.88 ± 0.3**	**	**5.31 ± 0.42**	**4.07 ± 0.41**	*
Brief awakenings ± SEM (events/hr)																		
All	**2 ± 0.14**	**1.49 ± 0.14**	*	1.4 ± 0.07	1.11 ± 0.13		2.49 ± 0.2	1.98 ± 0.18		**3.18 ± 0.16**	**2.49 ± 0.23**	*	**1.14 ± 0.11**	**0.56 ± 0.06**	***	**1.44 ± 0.11**	**1.07 ± 0.09**	*
Night	**1.95 ± 0.18**	**1.1 ± 0.13**	***	**1.02 ± 0.08**	**0.55 ± 0.09**	***	1.85 ± 0.2	1.52 ± 0.15		1.98 ± 0.14	1.52 ± 0.18		**0.92 ± 0.11**	**0.56 ± 0.06**	**	1 ± 0.14	0.66 ± 0.09	
Day	2.06 ± 0.16	1.88 ± 0.19		1.8 ± 0.1	1.68 ± 0.18		3.14 ± 0.27	2.45 ± 0.24		4.4 ± 0.25	3.47 ± 0.39		**1.37 ± 0.14**	**0.57 ± 0.08**	***	**1.95 ± 0.13**	**1.55 ± 0.13**	*
Wake ± SEM (min/hr)																		
All	17.0 ± 1.26	18.9 ± 1.5		18.8 ± 1.2	20.6 ± 1.4		13.1 ± 0.9	14.3 ± 1.2		20.0 ± 1.5	19.4 ± 1.4		14.5 ± 0.7	13.8 ± 0.8		**20.0 ± 1.3**	**16.4 ± 1.0**	*
Night	12.5 ± 1.2	14.8 ± 1.7		14.0 ± 1.4	17.0± 1.8		10.4 ± 1.1	10.5 ± 1.2		15.9 ± 1.6	14.3 ± 1.3		12.7 ± 0.8	11.8 ± 0.7		**18.2 ± 1.7**	**12.8 ± 1.2**	*
Day	21.4 ± 1.7	22.94 ± 1.9		23.8 ± 1.4	24.3 ± 1.5		15.8 ± 0.9	18.1 ± 1.5		24.0 ± 1.9	24.5 ± 2.4		16.5 ± 0.7	15.9 ± 1.1		22.1 ± 1.2	20.5 ± 0.9	
Mean sleep bout length ± SEM (min)																		
All	29.7 ± 2.8	33.8 ± 2.4		**26.8 ± 2.0**	**35.2 ± 2.5**	*	28.2 ± 3.1	37.1 ± 4.1		17.3 ± 1.3	28.4 ± 5.9		**19.9 ± 1.1**	**32.1 ± 2.0**	***	**21.4 ± 1.6**	**29.9 ± 2.7**	*
Night	34.9 ± 4.7	45.8 ± 4.2		**38.8 ± 4.6**	**57.1 ± 5.2**	*	37.3 ± 4.6	43.0 ± 4.9		**27.2 ± 2.5**	**47.0 ± 7.3**	**	**25.4± 2.3**	**41.9 ± 2.9**	***	**27.8 ± 3.3**	**43.9 ± 4.7**	**
Day	26.4 ± 2.5	26.5 ± 2.3		18.8 ± 1.3	22.5 ± 1.4		**22.4 ± 2.4**	**33.0 ± 4.3**	*	10.9 ± 0.8	18.1 ± 5.7		**16.0 ± 0.6**	**25.1 ± 1.7**	***	17.0 ± 0.87	21.0 ± 1.9	
Latency to sleep after light transition ± SEM (min)																		
Night	49.9 ± 3.8	60.5 ± 3.8		**26.6 ± 2.7**	**34.0 ± 2.1**	*	35.4± 5.6	25.7 ± 3.2		57.4 ± 7.2	58.5 ± 7.8		15.6 ± 1.4	18.4 ± 1.2		**50.5 ± 8.1**	**27.3 ± 3.2**	*
Day	98.4 ± 10.6	128.4 ± 17.1		55.4 ± 8.0	77.2 ± 13.6		**32.1± 5.9**	**82.2 ± 19.2**	*	**67 ± 10.3**	**132 ± 17.6**	**	20.2 ± 3.2	27.8 ± 4.7		49.6 ± 5.0	43.9 ± 5.5	
